# A Novel Approach to Data Collection for Difficult Structures: Data Management for Large Numbers of Crystals with the BLEND Software

**DOI:** 10.3390/cryst7080242

**Published:** 2017-08-04

**Authors:** Anastasia Mylona, Stephen Carr, Pierre Aller, Isabel Moraes, Richard Treisman, Gwyndaf Evans, James Foadi

**Affiliations:** 1 Signalling and Transcription Laboratory, Francis Crick Institute, 1 Midland Road, London NW1 1AT, UK; 3 Research Complex at Harwell, Rutherford Appleton Laboratory, Oxford OX11 0FA, UK; 4 Department of Biochemistry, University of Oxford, South Parks Road, Oxford OX1 3QU, UK; 5 Diamond Light Source Ltd., Harwell Science and Innovation Campus, Didcot OX11 0DE, UK

**Keywords:** BLEND, multiple crystals, hierarchical cluster analysis, protein-protein-DNA complex

## Abstract

The present article describes how to use the computer program *BLEND* to help assemble complete datasets for the solution of macromolecular structures, starting from partial or complete datasets, derived from data collection from multiple crystals. The program is demonstrated on more than two hundred X-ray diffraction datasets obtained from 50 crystals of a complex formed between the SRF transcription factor, its cognate DNA, and a peptide from the SRF cofactor MRTF-A. This structure is currently in the process of being fully solved. While full details of the structure are not yet available, the repeated application of *BLEND* on data from this structure, as they have become available, has made it possible to produce electron density maps clear enough to visualise the potential location of MRTF sequences.

## 1 Introduction

The current data collection and data processing landscape in X-ray crystallography for biomolecules is very different from the way it used to be even just 10 years ago. Several factors have contributed to this significant change, including improved technology at third-generation synchrotrons, faster readouts from silicon pixel detectors, ubiquitous presence of robotic arms and related cryogenics, new types of set-up for single and multiple crystal mounting, fast data transfer and larger capacity for data storage, new processing software and continued introduction and update of process pipelines. One of the noteworthy aspects of such an enhanced methodology is the assemblage of complete datasets from several crystals, as opposed to the acquisition of a complete dataset from one single crystal. A number of papers and related software [1–15] have appeared since 2011, the year in which Hendrickson and collaborators showed how datasets from multiple individual crystals could be merged to increase data multiplicity with the aim of reinforcing the anomalous signal due to heavy atoms [1]. The advantages of creating complete datasets out of several partial ones can be summarized as:

1. Increased likelihood of solving a structure even before having obtained well-diffracting crystals through the use of optimised crystallization conditions. This amounts to a significant saving in time, as many attempts are very often needed to find the right conditions that yield large crystals.
2. Increased data multiplicity, with the twofold consequence of obtaining better data scaling and stronger anomalous signal, if strong anomalous scatterers are present in the structure. A consequence of this so-called data redundancy is the recent finding that native proteins can be solved by exploiting the generally faint anomalous signal due to sulphur atoms, because such a signal is highly enhanced by the high data multiplicity [7,8,10,14,16,17].
3. More accurate structure factors. As scaled data are obtained merging individual observations from different, independent crystals, the derived structure factors might present larger errors, but better accuracy. Phasing and the resulting electron density maps, accordingly, have improved overall quality [3]. This qualitative observation holds if the different crystals have a reasonable level of isomorphism.
4. Physical limitation of the deteriorating effects due to radiation damage. Only the first portion of every dataset can be retained when merging data together, because later sweeps generally include reflections biased by the changing lattice, progressively altered by X-ray radiation.
5. New scenarios opened by the management of multiple datasets in relation to crystals isomorphism and structure dynamics. One such scenario is the use of multiple crystals for structure-guided drug design, whereby many crystals are soaked in a cocktail of chemical fragments that act as precursors for more complex drug molecules. Data is then collected from multiple crystals and merged to produce electron density maps that allow the identification of bound inhibitors [18,19].

A clear sign that use of multiple crystals has become an accepted methodology in the community of structural biologists is the setting up, at several synchrotrons around the world of technology, hardware and software [9,18,20–23] to make the technique routinely accessible to users, like, for example, the new in situ automated VMXi beamline [24]. Furthermore, the handling of large numbers of crystals for data collection is the standard mode of operation for free-electrons laser sources [4,5,13,25–27]. In fact, the free electron laser data collection paradigm has been successfully imported into 3rd generation synchrotrons [28–31].

While recent years have witnessed the effort to create adequate technologies for harvesting and processing large volumes of data in an automated fashion, a strong case still exists for manual handling of multi-crystal datasets. Although automated software has improved, with the advent of faster processors and large computing memories, the relevant routines are normally only successful with data that do not represent a high processing challenge. Macromolecular diffraction images that are difficult to interpret are periodically appearing in the community of interest, and the analysis of these same images is often used to improve automated software. In macromolecular crystallography, therefore, the need is still felt to manage and process data from multiple crystals, without resorting to automated programs. In this article, a computer program created for this purpose, *BLEND* [3], will be described with special focus on its use for handling data from the challenging structure of a complex between the Serum Response transcription Factor (SRF), one of its regulatory cofactors, and DNA containing an SRF binding site. This complex will be referred to as SRF-M-DNA throughout this paper, as its crystal structure has not yet been solved. The application of *BLEND* to data from this structure has enabled the creation of complete datasets of reasonable quality and provided us with procedural hindsight, useful when working with multiple datasets.

### Datasets from Single and Multiple Crystals

As previously mentioned, the final product of the assemblage of data from multiple crystals is a dataset that includes reflections from several distinct rotation sweeps. Such sweeps can be very wide, quite often covering the full unique portion of reciprocal space. Such datasets (i.e., those collected from a single crystal) will henceforth be referred to as *Dataset Single-crystal Complete* (DSC), and a dataset assembled from multiple crystals/smaller datasets, will be called *Dataset Multiple-crystals Complete* (DMC).

## 2 Collecting Data from More Than One Crystal: A Short Review

The main goal of data collection for macromolecular crystallographers is the measurement of reflection intensities with high completeness (close to 100%) and sufficient multiplicity. Completeness refers to that fraction of reciprocal space, up to a given resolution, sufficient to create an electron density map with minimal distortion. In general, completeness should be around 90% and above, but density maps have been calculated with lower values, and there are no rigorous criteria to suggest a resolution threshold to avoid map distortion. Multiplicity measures the average number of times all reflections have been measured, taking into account symmetry equivalence. Multiplicity should be at least 2 or better in order for scaling to yield accurate structure factors.

Until a few years ago, the preferred way to obtain complete datasets was by irradiating a single crystal with an attenuated X-ray beam while rotating the crystal through a large angle, quite often 360° or more. With the advent of 3rd generation synchrotrons, X-ray beams have become more powerful, so that data can be collected now from samples that before would not have provided enough scattering power for the detector to be triggered sensibly. But such intense pulses of photons damage the crystal irreversibly, and the sample's lattice is destroyed before data with enough completeness and/or multiplicity is obtained. The obvious way around this limitation is to collect scattered intensities from different individual crystals and assemble the diverse and often overlapping portions of reciprocal space into a single dataset having the required completeness and multiplicity (a DMC). A major problem with this approach lies in the heterogeneity of the different crystals, called in this context *crystal isomorphism.* Scaling the assemblage of individual datasets from different crystals can result in biased structure factors not representing the target structure, unless crystals have a good degree of isomorphism. Different ways of measuring crystal isomorphism can be imagined, and one of them will be explained when describing the *BLEND* program, but what is important when dealing with multiple crystals is that the more isomorphous the crystals are, the better and more accurate the quality of the structure factors will be. Two different approaches to the combination of multi-crystals are currently available. The first makes use of hierarchical cluster analysis (HCA) to create groups of datasets with a certain degree of similarity, as measured by various descriptors. To this group belong, among others, the procedure incorporated in the program *BEST* [2] and the program *BLEND* [3], described in the next section. The second approach starts from the full set of crystals available and proceeds towards a single, smaller group of crystals through a convergence process in which one or more crystals are discarded based on the decrease of a target function, typically an indicator of scaling quality, like Rmerge, Rmeas or, more recently, the CC_1/2_ correlation coefficient [32,33]. Other procedures can mix elements from each of the two approaches, depending on the goal to be achieved. Some of these involve the gradual inclusion of individual reflection within single or multiple datasets in a controlled way until a specific threshold has been reached or surpassed [34]. A last procedure makes use of local scaling techniques, anomalous signal optimization and dataset weighting to improve the anomalous phasing likelihood [15]. The success of these methods involves the exclusion or limitation of that portion of reflections mostly affected by radiation damage.

The rapid increase of structures solved using data from multiple crystals and the number of new technical arrangements at various synchrotrons' beamlines suggest that the construction of complete datasets using multiple crystals is becoming a viable alternative to single-crystal data collection and will, probably, very soon become the default choice in macromolecular crystallography.

## 3 The *BLEND* Program

**Figure 1:**
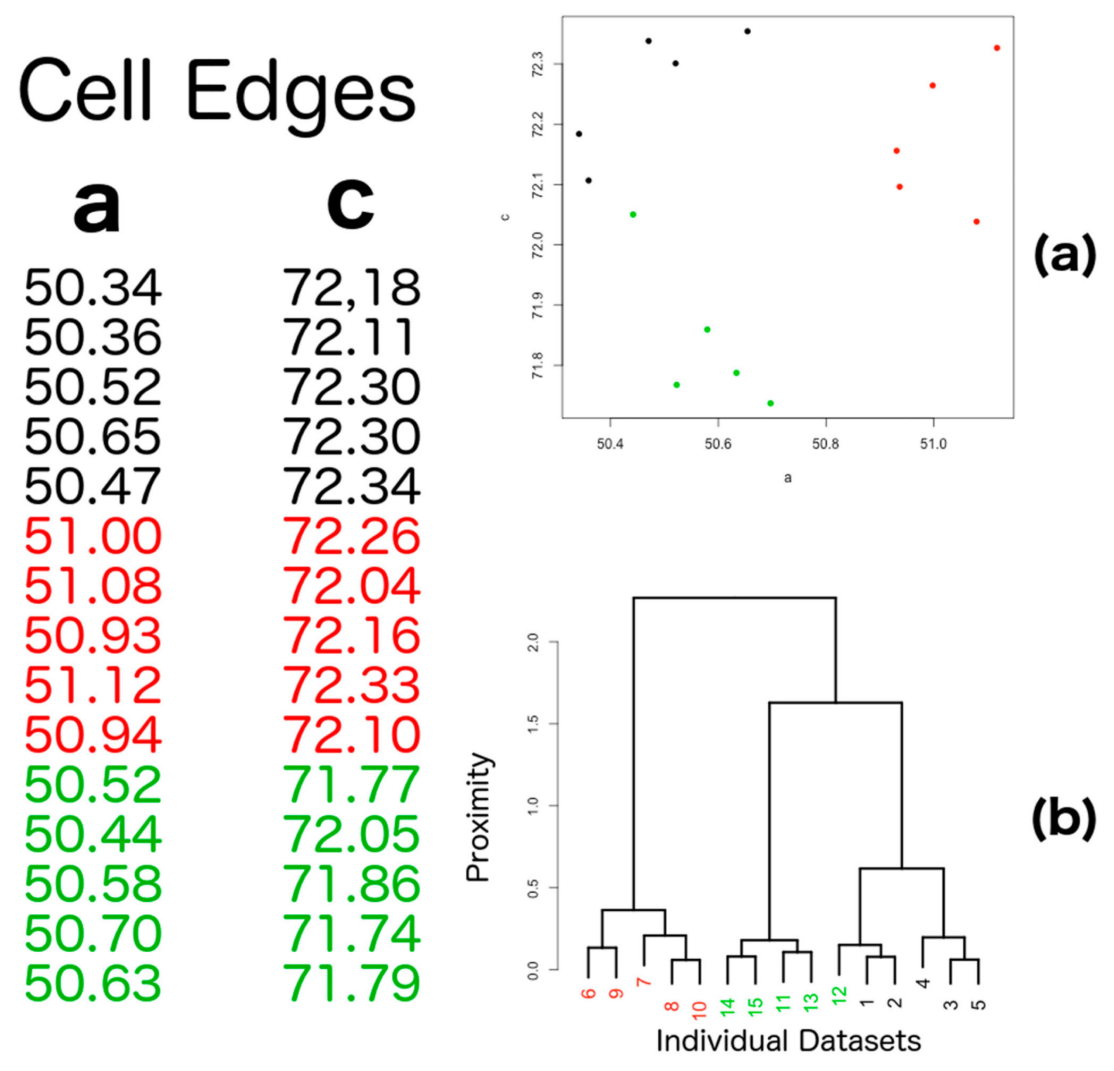
Hierarchical clustering, based on cell parameters for a simulated group of 15 crystals in a tetragonal space group. (**a**) Only lengths a and c of the unit cell are variable quantities useful to describe crystal variation in the tetragonal system (see data in panel on the left). The 15 crystals are separated in three groups (black, red and green) with similar structural features (crystal isomorphism). Cell parameters alone can be insufficient to discriminate among isomorphous groups. In this specific example, crystal 12 is closer to the black group than to the green group because the size of its unit cell is closer to the unit cell size of crystals 1 and 2; (**b**) Dendrogram reflecting hierarchical cluster analysis for the 15 crystals just described. The three isomorphous groups are well separated with the exception of crystal 12, forming a cluster with the black, rather than the green group.

The main purpose of *BLEND* is to provide guidance and tools for the merging of datasets from multiple crystals. The key ingredient of the program is hierarchical cluster analysis (HCA). This technique, developed within multivariate statistics, is very often used in initial data exploration to try and find connections, patterns and trends in datasets collected from independent sources. An old, but still effective, review of data clustering can be read in the long article by Jain et al [35]. In HCA, individual datasets are joined together into increasingly larger clusters based on their proximity. A distance between any two datasets can be defined once generalized coordinates are chosen that transform each dataset in a multi-dimensional point. These generalized coordinates are known, within multivariate statistics, as *statistical descriptors*. The primary statistical descriptors used in *BLEND* are related to the six cell parameters, i.e., the three cell edges, *a*, *b*, *c*, and the three angles, *α*, *β*, *γ*. Due to crystal symmetry, such descriptors can be as many as 6, in the triclinic system, and as little as 1, in the cubic system. Datasets are, thus, associated with numbers, and it becomes possible to measure the distance between each pair of them. In HCA, closer datasets will be grouped together first and joined later by other datasets or groups of datasets. The whole process gives rise to a tree-like structure, the *dendrogram,* in which individual datasets are like the tree's leaves, while clusters of increasing size are like the tree's branches that merge into bigger and bigger structures, eventually becoming the unique single tree's trunk (see Figure 1).

**Figure 2:**
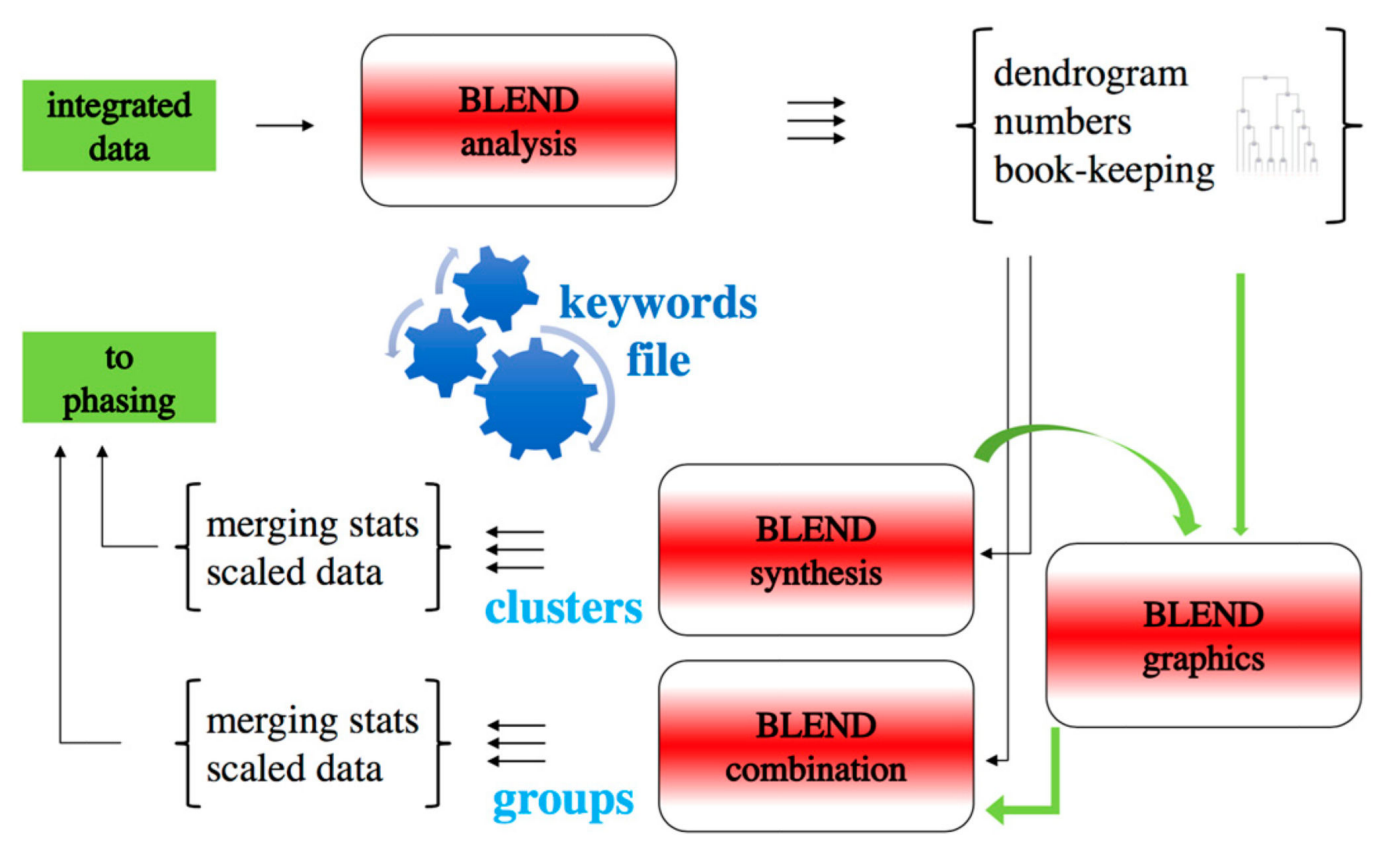
*BLEND* components and overall flow. The program can be executed in three main modes, *analysis, synthesis* and *combination.* Input are data from an integration program. After a run in analysis mode, the user has the option to re-run the program in synthesis or combination mode, in order to generate a given number of DMCs and DSCs. Other less important running modes are available, like the *graphics* mode, that are useful for in-depth data analysis. The various modes are controlled via keywords.

The creation of a dendrogram is, most of the time, meant to highlight homogeneous groups. In the specific process we are describing, the aim could be to single out one or more groups of isomorphous crystals. In *BLEND,* though, a different philosophy has been adopted since the very first version of the program. It is suggested that each cluster (each branching node in the tree) potentially leads to a useful solution. Subsequent processing of specific clusters, automatically carried out within *BLEND* using the programs *POINTLESS* and *AIMLESS* [36,37], reveals the sufficiency or inadequacy of the resulting dataset to be used for further processing leading to structure solution. There are many tools in *BLEND* to facilitate the analysis and further processing of each cluster. For this reason, the program can be considered semi-automated software, as it allows combined datasets to be assembled without human intervention, but requires synergy with the user for the definition of datasets with improved quality. There are many ways to create a DMC from different datasets after the initial clustering. Each way depends on the specific requirements of the final DMC. For example, if high resolution is required with the hope of observing side chains or even atom-atom bonds in the electron density map, then most of the constituent datasets with high resolution will need to be included, even if their merging statistics are not among the best available. Or, if a nearly-complete combined dataset still has not reached a desired target completeness, datasets with lower isomorphism can be included in the nearly-complete group, with the assumption that influence on the most important structural features in the electron density map will be negligible. Statistical quality indicators are produced by the software for each cluster or modified cluster. This makes it possible to carry out dataset creation and management according to users' preferences, guided by the quality indicators. The overall structure of the *BLEND* program with its various components is shown in Figure 2. There are, essentially, three different main running modes: (1) an analysis mode in which datasets are checked, information from each one of them extracted, and the dendrogram produced; (2) a synthesis mode in which datasets out of each cluster are combined and scaled; and (3) a combination mode, in which datasets not grouped in any existing cluster are combined and scaled. Other modes of execution also exist to allow additional types of operations, not possible within the remit of the three main modes. It is envisaged that more modes will be added in the future, and that eventually the program will be equipped with a graphical interface with which execution and interplay of the various modes will become more intuitive for users. Useful tutorials illustrating how to use *BLEND* with specific examples are available at the main CCP4 website [38]. A complete description of the many uses of *BLEND,* with special reference to membrane proteins is also available in “The Next Generation in Membrane Protein Structure Determination” [39].

### The Absolute Linear Cell Variation (aLCV)

**Figure 3:**
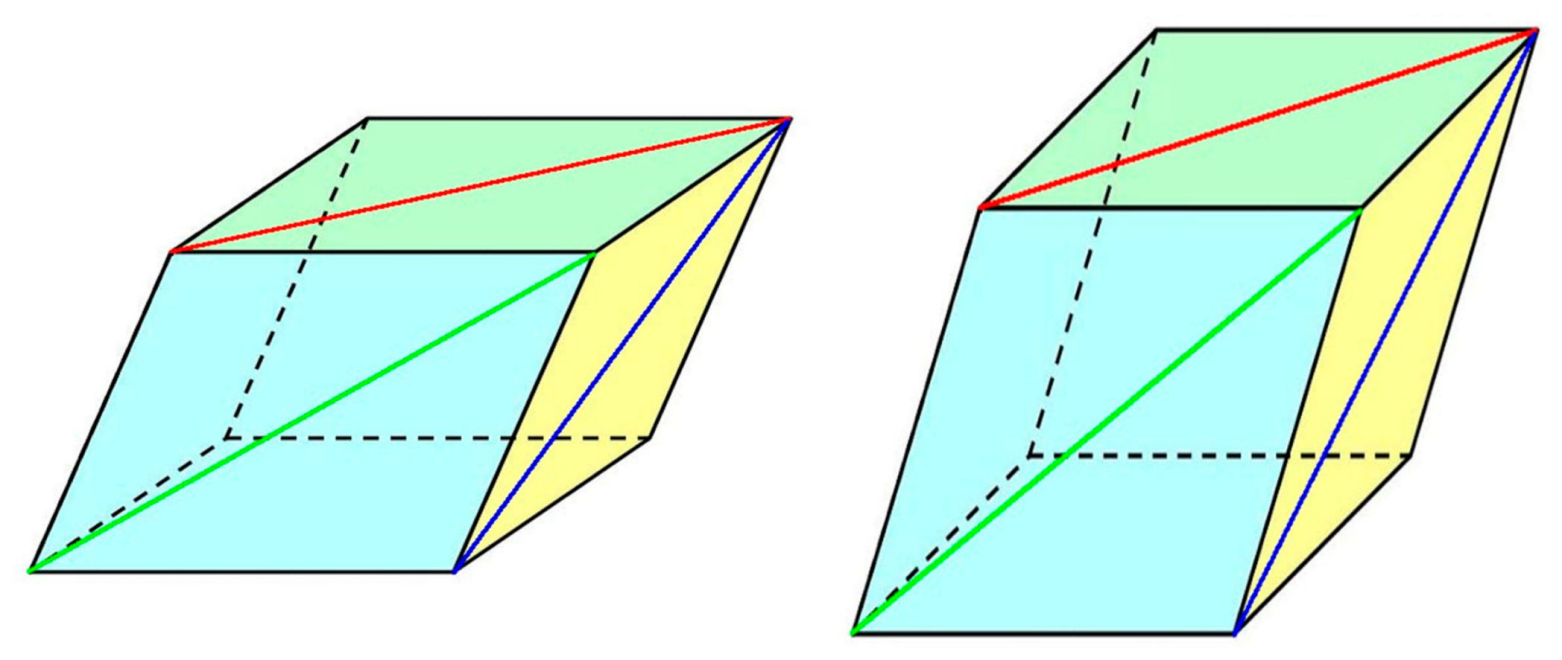
Meaning of aLCV. Two generic unit cells are represented in this figure. The three diagonals along each one of the three main unit cell's faces depend both on the cell's sides and the cell's angles. Any of the diagonals in one of the two cells will be, in general, different from the corresponding diagonals in the other cell. Let's call Δa, Δb, Δc the difference between corresponding diagonals. The aLCV (absolute Linear Cell Variation) for the group formed by the two unit cells is the maximum difference: aLCV = max(Δa, Δb, Δc). When more crystals are added, the aLCV is recalculated as before, considering all pairs of unit cells, and selecting the highest of all maximum values computed as the new aLCV.

In order to provide users with a single number describing unit cell isomorphism, a new parameter has been introduced in *BLEND.* This is called *absolute Linear Cell Variation* (aLCV) and the way it is defined is shown in Figure 3.

In Figure 3, the simple case of two unit cells is shown. The variation of one cell with respect to the other can be due to both the three sides and the three angles. Thit is what *BLEND* uses when calculating cluster analysis. The height reported in the corresponding dendrogram, though, is not related to any absolute difference in linear or angular measurements between the unit cells involved in the dendrogram. This is where the aLCV plays a role. Consider any of the three diagonals on the three main faces of each unit cell. The diagonal is measured in angstroms, and its variation is due to both the cell's side and angle variations, simultaneously. The difference between corresponding diagonals for the main unit cell's three faces are the numeric values in angstroms: Δa, Δb, Δc. The aLCV for the two crystals under consideration is the maximum among the three numeric values:

(1) aLCV=max(Δa,Δb,Δc)

When more than two crystals are used, quantity (1) will be calculated between all couples of unit cells in the group, and the aLCV will be equal to the highest value obtained. The aLCV parameter is, accordingly, measured in angstroms.

## 4 Materials and Methods

To illustrate how *BLEND* can manage datasets from multiple crystals for the creation of one or more DMCs, we have chosen to describe work done with 271 datasets from 50 crystals, collected during 7 sessions at the Diamond Light Source synchrotron [40]. Full details are included in Table A1, Appendix A.

### 4.1 The Target Structure

The crystal structure that prompted the investigations described in this paper is a multicomponent complex comprising the DNA-binding domain of the SRF transcription factor, bound to its cognate DNA and a synthetic peptide from the SRF cofactor Myocardin Related Transcription Factor (MRTF-A—referred to hereafter as SRF-M-DNA). SRF controls growth factor-inducible, cytoskeletal, and muscle-specific genes by recruiting members of two families of signal-regulated transcriptional coactivators, the MRTFs and the Ternary Complex Factors (TCFs), which interact with its DNA-binding domain [41,42]. Structural studies have extensively characterized the interaction between SRF and DNA, and its interaction between SRF and the SRF Accessory Protein (SAP-1 TCF) [43,44]. However, while biochemical studies show that the MRTFs and TCFs compete for a common surface on the SRF DNA-binding domain [45], the structural basis of the MRTF-SRF interaction remains to be determined. In this project, we thus sought to define the interaction between MRTF and SRF, and to elucidate the nature of any interaction between MRTF and the DNA. A major challenge has been to obtain a low-resolution image of the complex, which includes a long and flexible DNA fragment, and indeed, the best resolution obtained to date has been between 3.5 and 4 Å. We used *BLEND* processing to combine different SRF-M-DNA datasets in sensible ways, which has allowed us to generate a low-resolution image of the SRF-M-DNA interaction.

### 4.2 Data Collection and Plans to Solve the Structure

Once the first crystals were obtained and X-ray data collected, it became apparent that the resolution was limited. Complete, single datasets were used to try and phase the structure using molecular replacement with SRF as a partial model. All attempts were unsatisfactory and it was, subsequently, decided to use combined datasets in the hope of obtaining interpretable electron density maps. *BLEND* was executed numerous times on an increasing number of datasets until a promising DMC could be assembled. The resulting map showed density corresponding to the SRF part and of some DNA, but it was very noisy and did not convincingly show MRTF density. We decided to collect more data from newly grown crystals, both using more crystals of the same type, and trying different data collection strategies, to test whether BLEND could yield further DMCs, with the aim of producing more interpretable electron density maps. Unfortunately, the addition of new datasets did not generate better maps. The reason was related to map isomorphism: datasets corresponding to similar unit cells can potentially describe structures that are not very isomorphous, which can hinder calculation of electron density of good quality. Furthermore, when the number of datasets forming a dendrogram is too high, it becomes more difficult to carry out the filtering and combination of separate clusters and groups, because the possibilities are, in this case, endless. We therefore decided to approach the processing and management of all datasets collected in a more systematic way, as described in the next section.

### 4.3 Pre-Clustering

As presently structured, BLEND discriminates datasets based only on the chosen statistical descriptors. If we stick with unit cell parameters for now, it is clear that one dataset will be different from another according to the similarity of their unit cells. But two datasets with identical unit cells can still be different for many reasons. They could correspond to distinct structures (unlikely, but theoretically possible); they could come from crystals grown in different conditions; they could have been collected during different visits, and so on. The original philosophy in BLEND was to ignore differences with the exception of those leading to clustering. But recently it has been found that separation of data in groups, prior to clustering, can help save processing time later, because it reduces the number of possible clusters.

In this paper, the division of all datasets could have been carried out in many ways. It is quite sensible, for example, to think that datasets corresponding to crystals prepared with the same crystallization and cryogenic conditions, and containing the same heavy atom, would tend to be fairly isomorphous and should, then, be separated in a single group prior to clustering. Another sensible choice would be to create a group of datasets corresponding to the same heavy atom, even though the crystals were prepared with different crystallisation procedures; in this case the heavy atom is thought to influence isomorphism to a greater extent than other factors. Whichever the criteria employed to effect an initial separation into groups are, it is important to have an algorithmic structure that makes the separation easy. Such a structure has not been yet coded within *BLEND,* but it has been used for the work in this article and will now be described.

The starting point is the raw data listed in Table A1. This table can be encoded in a dataframe, in the context of the R programming language [46]. A dataframe is, very simply, a table with columns and rows. The way it is encoded in the R language means that it can be reshaped into other objects containing the same information as the original dataframe, but highlighting specific details. The main outcome of reshaping the original dataframe included in Appendix A is the creation of a new table, Table 1, in which each row corresponds to a unique combination of base conditions (BC), cryogenic conditions (CC), dehydration protocol (DH), flag (yes, no) indicating whether the heavy atom was co-crystallised (CO) and heavy atom type (HA). The construction of this table is connected to the creation of a new dataframe, the conditions dataframe, explained in Appendix B.

The most useful feature of this dataframe is that it makes it immediately clear how many datasets are available for the specific combination of crystal features. For example, the largest number of datasets (59) is found in the group with serial number 13. Crystals in this group were grown with base condition bc1, dehydrated with protocol dh1, incorporated a platinum atom via soaking with a solution of K_2_PtCl_4_, and were cryo-cooled after being prepared with condition cry1.

### 4.4 Strategy for Data Combination

As the goal of the approach chosen in our investigations was to find out if electron density maps clearly displayed the interaction of MRTF with DNA, and possibly with SRF, we decided to create data starting from the groups with the highest number of datasets, because these were more likely to yield more DMCs. The research that will eventually lead to the solution of SRF-M-DNA is still ongoing, as not all datasets collected have been properly explored. Up until now, the groups that have been explored and used in this paper are serial group 13 (59 datasets), serial group 27 (42 datasets), serial group 25 (28 datasets), serial group 16 (24 datasets), serial 14 (23 datasets) and serial 2 (14 datasets). From each group, one or more DMCs were assembled and used to try and solve the structure. Only work done on two of these groups, serial group 25 and serial group 2, will be described here in detail, because they are the only ones that have so far been used to calculate interpretable electron density maps. It is clear that many more combinations of the many datasets available could be considered for further work.

### 4.5 Detailed Description

#### 4.5.1 Working out DMCs with Serial Group 25

**Figure 4:**
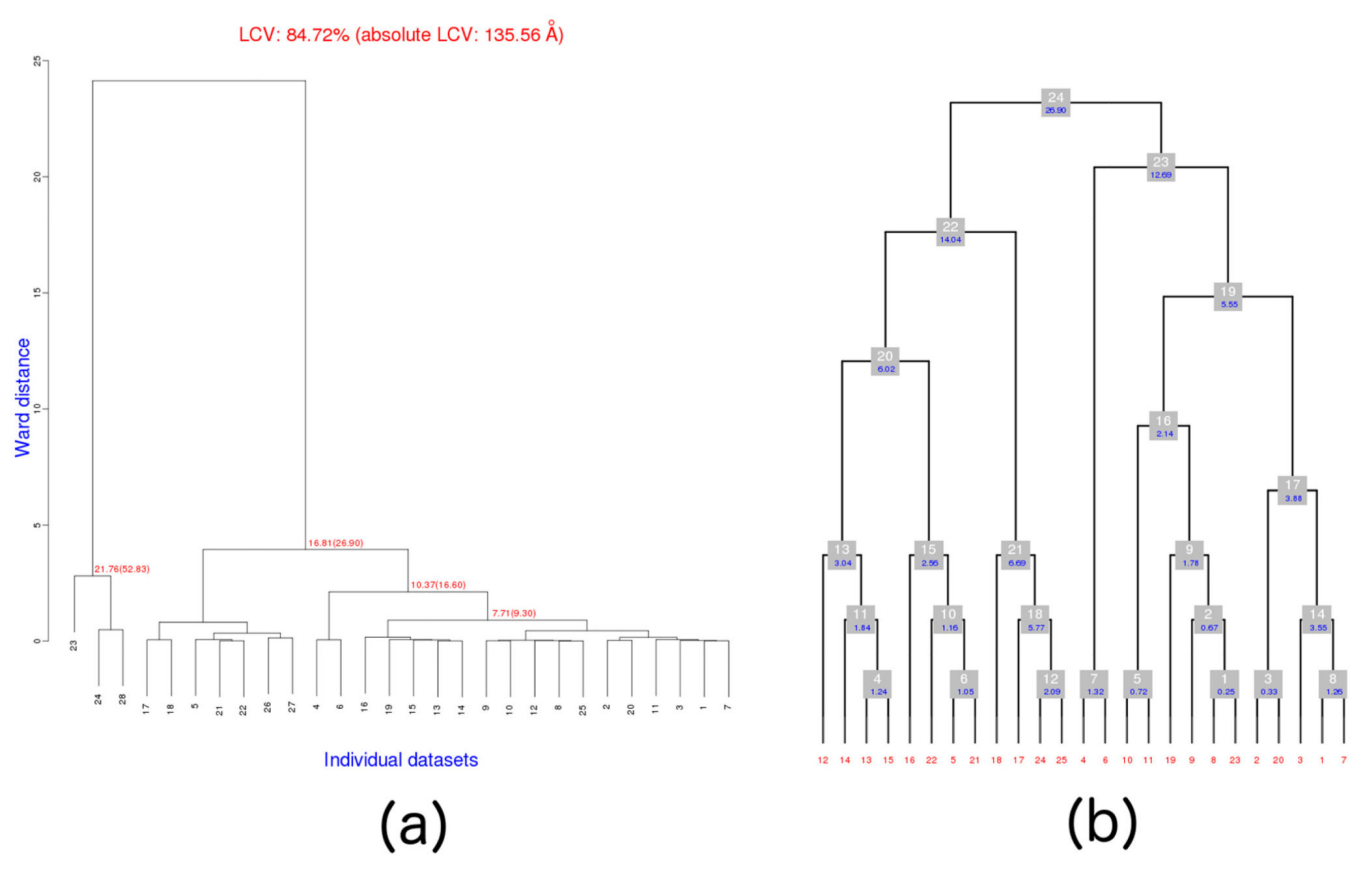
Dendrograms corresponding to runs of *BLEND* on datasets of serial group 25. **(a)** Initial run on all 28 datasets. This is the default dendrogram produced by *BLEND* when executed in analysis or dendrogram-only mode. In here it is immediately clear that datasets 23, 24 and 28 are outliers; **(b)** Execution of BLEND on the remaining 25 datasets. This dendrogram has a different style, compared to that in part **(a),** with no cluster height but, rather, cluster level, where nodes at each level have the same number of objects.

In this group, there are 28 datasets in space group P222_1_, collected from 10 crystals formed with a solution containing gadolinium, subsequently soaked in a solution containing osmium, and finally dehydrated with the addition of salts corresponding to protocol dh1. The best strategy with *BLEND* at the very beginning of a data combination process is to execute the program in *dendrogram-only* mode (option –aDO) in order to check the validity of all files involved, and to display the dendrogram. The program does not produce all files needed for subsequent runs, but it runs faster in this mode, and rapidly identifies outliers among the datasets. The result of the analysis of all 28 datasets in group 25 is shown in Figure 4a, and it is clear that datasets 23, 24 and 28 are very non-isomorphous with the other datasets and also among themselves.

For this reason, it makes sense to consider them as outliers and re-run *BLEND* on the remaining 25 datasets. The results of the second run are shown in Figure 4b. This figure was obtained with the help of *BLEND's* graphics mode (option –g), executed after the dendrogram-only mode. From the figure, it is easy to appreciate that the dendrogram is not displayed using a cluster's height but, rather, using the number of objects included in a cluster. So, for instance, the first level of grey boxes corresponds to clusters of 2 objects; the second level corresponds to clusters of three objects; the third level corresponds to clusters of four objects, and so on. This type of dendrogram representation in *BLEND* also includes, for each node, information on cell isomorphism (the parameter aLCV) and cluster number. It is important to observe that the 25 remaining datasets used for the second run of *BLEND* were renumbered from 1 to 25 so that the original numbering was lost in the second run. One of the files produced by *BLEND,* “FINAL_list_of_files.dat”, includes information on all datasets used. From this file, it was clear that the first seven datasets were wider and more complete sweeps, compared to the remaining 18. A very crude resolution estimate was also computed by *BLEND* and recorded in “FINAL_list_of_files.dat”. For the 25 files treated, these estimates ranged roughly between 2.7 Å and 4.7 Å. For the following runs it was, therefore, decided to treat the 7 complete datasets separately from the 18 partial sweeps. Furthermore, for the merging and scaling steps within *BLEND* synthesis or combination, the resolution was arbitrarily set to 4 Å, both based on experience applying *BLEND* to other structures and because this resolution is situated between the estimated highest and lowest resolutions. Scaled data for the 7 complete datasets could be obtained by executing *BLEND* in combination mode with the following syntax:

blend-c[datasetserialnumber]

With “dataset serial number” being the serial number associated with any of the first seven datasets of the run with 25 datasets. Statistics for the 7 complete datasets are displayed at Table 2.

By far, the best solution is the one associated with dataset 7, which was selected as one of the DSC to be used for phasing. In the hope of extending resolution to 3.5 Å, *BLEND* combination was run again on dataset 7 with the keyword RESO HIGH 3.5. Unfortunately, the overall Rmeas deteriorated substantially and, in the end, it was decided to extend resolution to just 3.8 Å.

**Figure 5:**
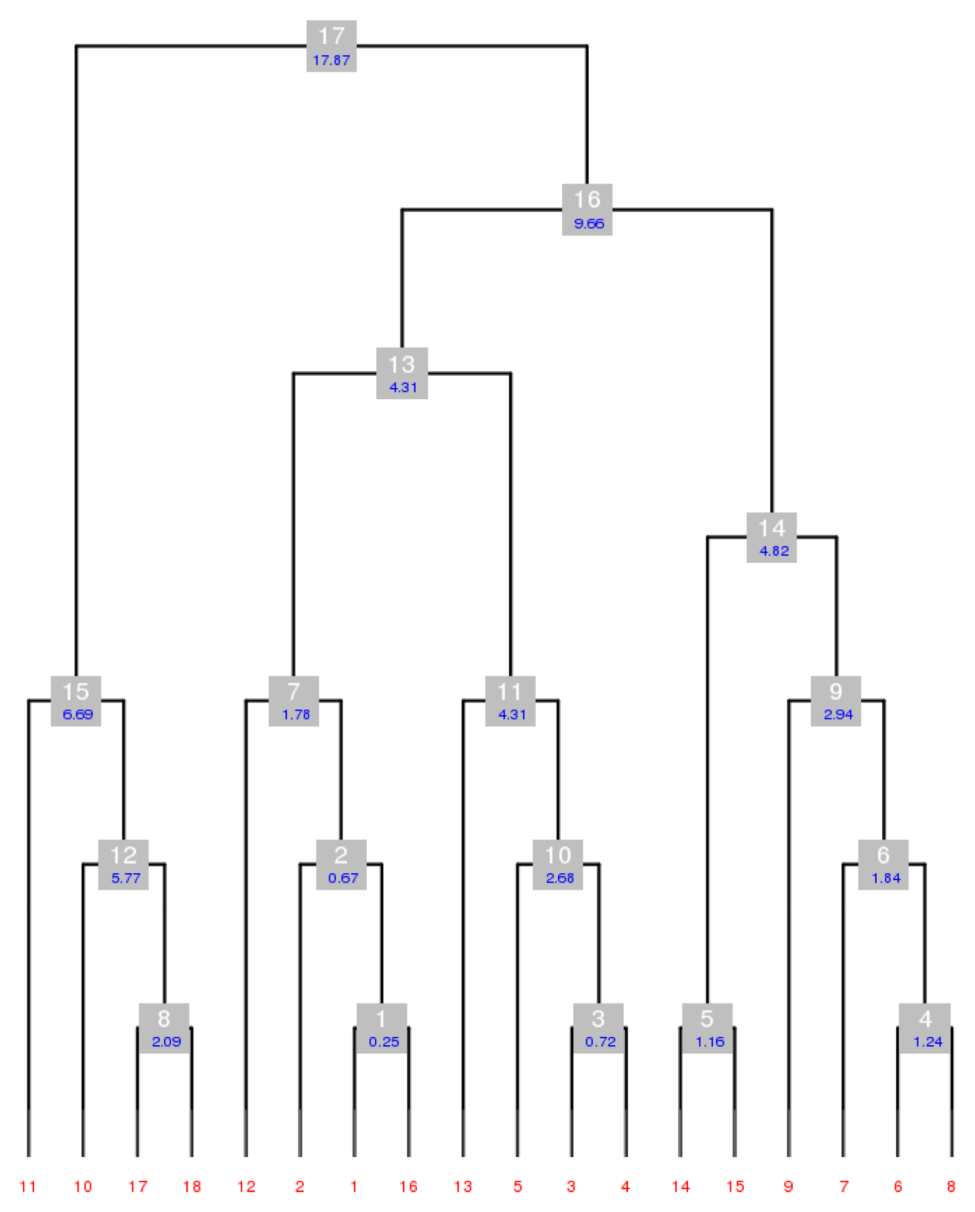
Dendrogram for the 18 partial datasets in group serial 25.

In order to execute *BLEND* on the 18 remaining and partial datasets, it was necessary to re-run the program in analysis mode using only these 18 datasets. The resulting dendrogram is displayed at Figure 5 (dataset numbers are, once more, changed for this run; now running from 1 to 18).

To see how well the 17 clusters produced performed with scaling, we ran *BLEND* on each one of them in synthesis mode (maximum resolution fixed at 4 Å). The resulting statistics for data with completeness greater than 90% are collected in Table 3.

From the table, it is clear that cluster 7 displayed much better merging statistics, which agrees with the relatively low value for aLCV (1.78 Å), but the completeness is not close to the ideal value of 100%; therefore, using the dendrogram in Figure 5, it was decided to consider a large cluster that included cluster 7 as a starting point for the automated filtering variant within the combination mode in *BLEND.* In this variant, one dataset at a time is discarded from a starting group of datasets until convergence towards a low Rmeas is achieved, provided completeness remains above a specified threshold level. When *BLEND* was executed in combination mode with this variant, and starting from cluster 13, only one dataset was automatically discarded, and the final completeness reached 99.3%, Rmeas equaled 0.310, Rpim equaled 0.118 and the multiplicity reached 6.6.

So far, from serial group 25, one DSC and one DMC were selected for further work with structure solution: dataset 7 (see Table 2) was the selected DSC, while cluster 7 (see Table 3) was the selected DMC.

#### 4.5.2 Obtaining DMCs from Serial Group 2

**Figure 6:**
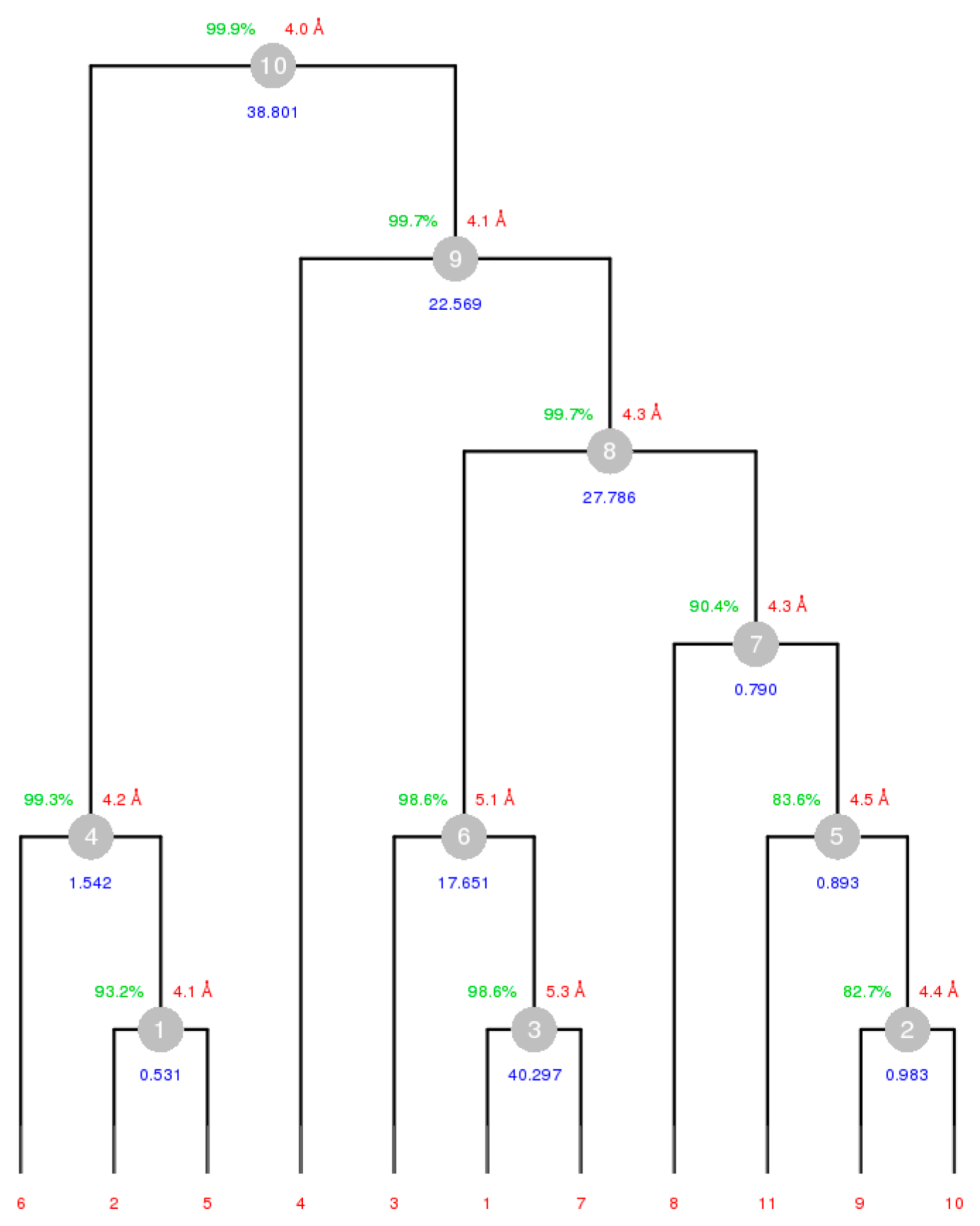
Annotated dendrogram for the 11 datasets used from the serial group 2. Each numbered node corresponds to a cluster Around each node there are three values annotated with overall completeness (green), resolution as computed using CC_1/2_ (red) and overall Rmeas (blue).

Work to obtain complete datasets (also with P222_1_ symmetry) from this other group followed a similar pattern to what was described for serial group 25. *BLEND* (dendrogram-only mode) was executed on the 14 datasets composing this serial group. Three outliers were found (datasets 12, 13 and 14) based on the comparison of aLCV, and discarded from the analysis. Next, *BLEND* was executed in full analysis mode on the remaining 11 datasets, followed by a run in synthesis mode, with resolution 3 Å, on all the 10 clusters obtained. The result is depicted in the annotated dendrogram of Figure 6. Each cluster corresponds to a numbered grey disc. Around each disc are located three numeric values corresponding to: (a) completeness (in green); (b) resolution as calculated from CC_1/2_ (in red); and (c) Rmeas value (in blue). Clusters 4, 6, 8, 9 and 10 have high completeness, but poor merging statistics. Therefore, the filtering variant of the combination mode was applied to these clusters, in the hope of improving the statistics. Results of the five runs of *BLEND* are reported in Table 4.

Values of Rmeas and Rpim are still a bit high, and the resolution estimates reported are closer to 4 Å than to 3 Å. It was decided to lower the resolution with the hope of obtaining more reasonable statistics. Also, it is interesting to observe that the obtained values for the third and fourth row are identical; this is to be expected because cluster 8 without dataset 7 coincides with cluster 9 without datasets 4 and 7. Results from the run at resolution 3.5 Å are included in Table 5.

The improvement derived from cutting the resolution to 3.5 Å is evident when looking at the new statistics, and illustrates a common trait when dealing with multiple data sets. That is, multiplicity and, in part, completeness are sacrificed in order to improve data scaling (measured by Rmeas and Rpim). The removal of data at unrealistically-high resolutions is also common practice, whose likely effect is to improve data quality, since data at high resolutions contain often more noise than signal.

To improve the quality of scaled data even more, we investigated the effects of a further source of noise, radiation damage. Reflections included in the last images of the rotation sweep are likely to reflect a structure substantially changed by the destructive power of energetic X-rays. Accordingly, such reflections are likely to be systematically different from those in the initial images which correspond to the non-damaged structure. So, when statistics are poor and the resolution has already been limited it may be desirable to exclude the final images of each dataset (especially, when it is evident that substantial radiation damage has occurred) in order to improve data quality. In *BLEND,* this can be done manually, using the BATCH EXCLUDE keyword or automatically as a variant of combination mode, the *pruning* variant. When *BLEND* is executed with this variant, there is an automated assessment of how many images can be eliminated without affecting threshold completeness. Based on this, images are cyclically eliminated from scaling until the threshold completeness is reached, or until the best scaling statistics have been achieved. This variant has been attempted on all filtered clusters of Table 5, resulting in a further improvement in data quality (Table 6).

All statistics have, in general, improved. Values for cluster 10 in Table 6 are unchanged, because the automated pruning procedure has not eliminated any images. The filtered dataset described in the last row of Table 5 and the filtered and pruned dataset described in the first row of Table 6, are the best DMCs with which to attempt structure solution, for data in serial group 2.

### 4.6 Structure Solution

#### 4.6.1 Data Used

Two of the four datasets prepared with *BLEND* have been used to attempt structure solution so far. These are the DSC mentioned in Section 4.5.1 and the DMC from Table 5. We will call the first dataset “serial25_01.mtz” and the second “serial02_01.mtz”.

#### 4.6.2 Molecular Replacement

The structure of the SRF part of the macromolecular complex has been previously solved in a different context. Therefore, one of the molecules of a structural complex published in the PDB repository [47], code 1HBX, and a shortened part of the DNA segment associated with the structure, were used as initial models for molecular replacement, in order to calculate initial phase estimates for our structure. Molecular replacement was performed using *PHASER* [48]. With dataset “serial25_01.mtz”, *PHASER* found a solution with Z-score for the translation function (TFZ) equal to 16.5. For dataset “serial02_01.mtz”, *PHASER* found a solution with TFZ = 16.2. As TFZ with values greater than 8 are declared to correspond to correct solutions, it is evident that both datasets have been assigned promising initial phases, and that SRF is a stable component of the complex.

An important result to verify was the accordance of the solutions found. The model for SRF is placed with a certain orientation at a specific location in the unit cell, which can be different for each of the two datasets. If the crystals used to produce the datasets are isomorphous and correspond to the same structure, then the two solutions found with *PHASER* should return the model with the same orientation and located in the same region of the unit cell. In simpler terms, the two models should overlap after molecular replacement. This was found to be the case: after molecular replacement, the RMSD between the two models, across all atoms, was 0.773 Å. The procedure is explained in Appendix C. In fact, for all datasets tried so far (data not included in this paper), the models found have proved to overlap very well.

#### 4.6.3 Structure Refinement and Electron Density

Initial models were extended using *COOT* [49]. The electron density showed enough structural details for the addition of DNA, starting from the short segment included in the initial model. After a few cycles alternating model building and refinement using the program *REFMAC* [50], most DNA could be built. In addition, clear protein electron density was visible in a region around the SRF component. Poly-alanine models could be built in this region for both datasets. Resolution range, completeness and overall refinement statistics for both models can be found in Table 7.

**Figure 7:**
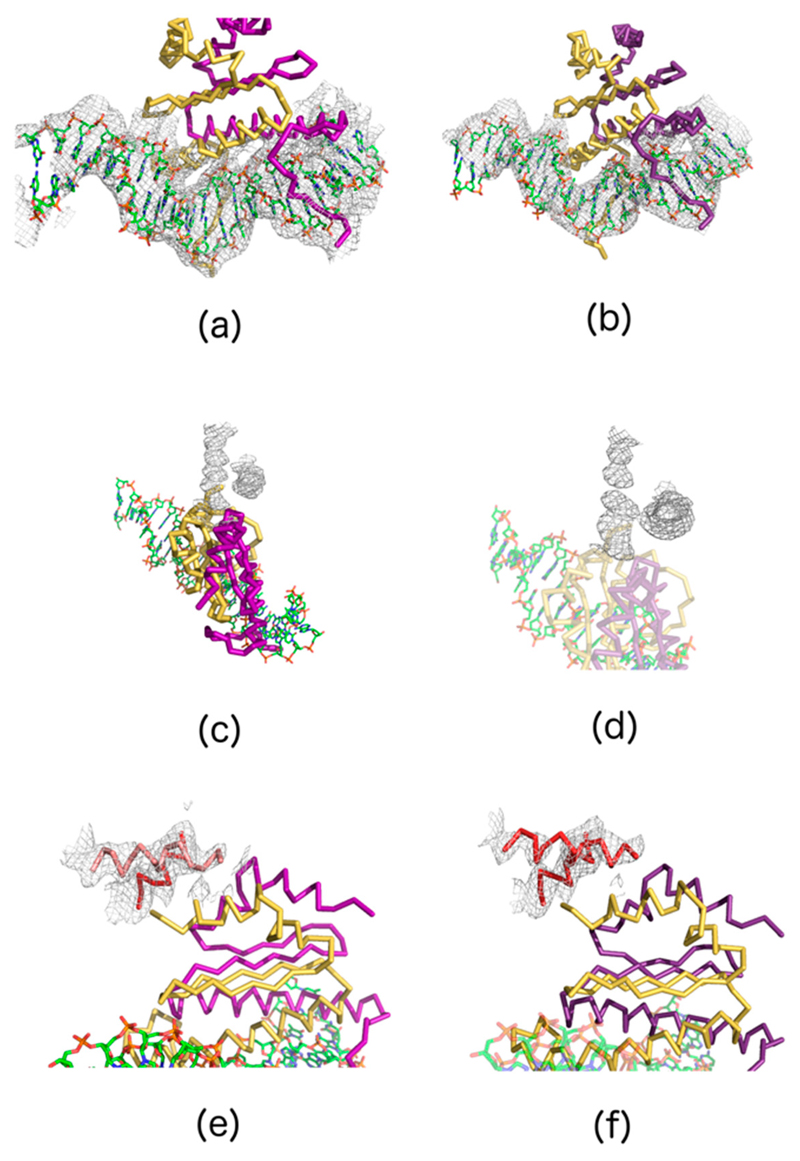
Details of the model and the electron density for the two datasets used in this paper. Figures **(a,c,e)** correspond to dataset “serial25_01.mtz”; figures **(b,d,f)** correspond to dataset “serial02_01.mtz”. The two top figures **(a,b)** display the quality and extent to which the DNA has been built. Figures **(c,d)** in the middle show details of the electron density presumably corresponding to the MRTF peptide which closely approaches SRF in the complex. The MRTF density had sufficient detail to allow fitting of an incomplete poly-alanine model **(e,f).**

The observed values reflect that the model is still very incomplete, the resolution limited and because the DNA confers some flexibility to the overall structure. Nonetheless, two goals have already been achieved with the two datasets used: (1) much of DNA structure could be built and fitted in clear density; and (2) additional protein-like density that could not be ascribed to SRF is visible, indicating the possible location of MRTF-A for the first time. Model and electron density details are shown in Figure 7.

## 5 Discussion and Concluding Remarks

While the priority in working with multiple datasets is the acquisition of a DMC that can be used to start the structure solution process, it is clear that the abundance of data involved can be used to increase the information on the biological problem under investigation. Crystal structures are formed by molecules that are stuck in unnatural positions and orientations because of lattice constraints. Each macromolecular crystal packs the molecules in a slight different way so that the electron density due to the X-ray diffraction from the crystal is an averaged and slightly blurred representation of the crystallised macromolecule. With multiple crystals assembled to produce a DMC, the blurring is more accentuated, especially when crystal isomorphism increases. Therefore, the process of grouping together crystals that are more likely to be isomorphous, as it is the case with *BLEND,* minimises such blurring and highlights conformational differences among non-isomorphous groups.

With the SRF-M-DNA complex analysed here, the situation is somewhat different because the resolution and the molecular dynamics do not make it easy to produce an electron density map of sufficient quality to appreciate molecular details. The priority in the first stage of the investigations of the SRF-M-DNA structure was to determine the overall shape of the complex and where MRTF binds to SRF. The ability to combine so many different datasets in a systematic and rational way, using a flexible tool like *BLEND,* offers valuable insights in this approach to structure solution. Several DMCs were used independently with the same initial model to produce molecular replacement solutions. All the solutions were consistent with the position and orientation of both SRF and the DNA. This, obviously, reinforces trust in the overall architecture suggested for the solution. In statistical terms, the different crystals can be seen as independent sources of information and the overlapping nature of the corresponding independent models points to an objective structure solution.

The SRF-M-DNA structure will now allow us to undertake a more detailed analysis of potential MRTF-SRF interactions. It is difficult to formulate a final judgment on the locations of the putative MRTF-SRF interaction seen in the current crystal model, especially in relation to previous biochemical analyses [45]. The low resolution of the diffraction data means the structure currently gives limited insight into the details of MRTF-DNA interactions, as yet. Further refinement of the structure, and additional biochemical analyses, will be required to resolve these issues. However, the consistency with the molecular replacement result, and the availability of many more datasets and potential combinations make us optimistic that additional density can be revealed in the map. Nevertheless, this study shows that systematic use of multiple crystals can substantially advance structural investigations in which straightforward and traditional approaches are not feasible.

## Supplementary Material

Appendix

## Figures and Tables

**Table 1:** Conditions dataframe, the tool used to determine the starting groups on which *BLEND* was executed. BC stands for base conditions in which the crystal was grown, CC for cryogenic conditions, DH for the type of dehydration procedure used, CO is a yes-no flag stating whether the heavy atom derivative comes from a co-crystallisation, and HA is the type of heavy atom. The dataframe also includes a column to assign a serial number (SN) to the specific group, and a column (NC) indicating the number of datasets for that specific group.

BC	CC	DH	CO	HA	NC	SN
bc1	cry2	no	no	no	13	1
bc1	cry1	no	no	no	14	2
bc2	cry1	no	no	no	5	3
bc1	cry1	dh1	no	no	7	4
bc1	cry1	dh1	no	KlCl_6_	6	5
bc1	cry1	dh1	no	Tantalum	1	6
bc1	cry1	dh1	no	Hg(Thi)	3	7
bc1	cry1	dh1	no	Pt(PIP)	1	8
bc1	cry1	dh1	yes	Pt(PIP)	1	9
bc1	cry1	dh1	no	KAu(CN)_2_	3	10
bc1	cry1	dh1	yes	KAu(CN)_2_	1	11
bc1	cry1	dh1	no	Hg(Ace)	1	12
bc1	cry1	dh1	no	K_2_PtCl_4_	59	13
bc3	cry1	dh1	no	K_2_PtCl_4_	23	14
bc1	cry1	dh2	no	K_2_PtCl_4_	4	15
bc1	cry3	dh2	no	K_2_PtCl_4_	24	16
bc1	cry1	dh1	yes	K_2_PtCl_4_	4	17
bc1	cry1	dh1	no	Hg(PMA)	1	18
bc1	cry1	dh1	no	K_2_PtI_6_	1	19
bc1	cry1	dh1	yes	OsCl_3_	1	20
bc1	cry1	dh1	yes	AgN	1	21
bc1	cry1	dh1	yes	I3C(magic triangle)	1	22
bc1	cry1	dh1	yes	GdCl_3_	9	23
bc1	cry1	dh1	no	Os	5	24
bc3	cry1	dh1	no	Os	28	25
bc1	cry1	dh2	no	Os	11	26
bc3	cry1	dh2	no	Os	42	27

**Table 2:** Scaling statistics for the 7 complete individual datasets of serial group 25. Maximum resolution is fixed at 4Å from suggestions based on the *BLEND* analysis run. The best results in this group of 7 datasets point to dataset n. 7 (last two rows).

Dataset Number	Rmeas	Rpim	Completeness (%)	Multi-Plicity	Resolution CC1/2	Resolution Mn(I/sd)	Resolution Max
1	0.472	0.217	93.5	3.8	4.39	5.82	4.00
2	0.537	0.298	92.3	2.7	4.97	5.70	4.00
3	2.107	1.430	97.9	2.6	4.00	4.00	4.00
4	0.328	0.137	99.9	6.5	5.85	5.93	4.00
5	0.532	0.311	78.7	2.8	6.21	6.57	4.00
6	1.510	0.788	71.5	3.5	5.77	6.18	4.00
7	0.212	0.104	99.9	6.4	4.08	4.37	4.00
7 final dataset	0.277	0.112	98.9	4.4	3.80	4.39	3.80

**Table 3:** Scaling statistics for clusters in serial group 25 reaching data completeness of 90% and above. Cluster 7 seems promising, but its completeness has to be increased. This was done (see text) using the filtering variant of the combination mode.

Cluster Number	Rmeas	Rpim	Completeness (%)	Multi-Plicity	Resolution (CC1/2 = 0.3)	Resolution (Mn(I/sd) = 2)	Resolution Max
14	0.979	0.398	99.9	6.2	4.91	5.04	4.00
16	0.958	0.260	99.8	13.7	4.24	4.36	4.00
17	1.758	0.437	99.7	16.1	4.27	4.35	4.00
9	0.778	0.377	99.5	4.2	4.92	5.62	4.00
13	0.618	0.210	99.2	7.6	4.00	4.37	4.00
11	0.632	0.290	97.9	4.3	5.20	4.70	4.00
10	0.707	0.377	97.2	3.2	5.41	5.11	4.00
7	0.337	0.158	92.2	3.7	4.00	4.58	4.00

**Table 4:** *BLEND* run in combination mode with the filtering variant, for the 5 most complete clusters in the serial group 2case.

Cluster Number	Datasets Filtered	Rmeas	Rpim	Completeness (%)	Multi-Plicity	Resolution CC1/2	Resolution Mn(I/sd)	Resolution Max
4	6	1.542	0.544	99.3	8.5	4.25	4.86	3.00
6	none	17.651	7.004	98.6	7.0	5.13	6.26	3.00
8	7	9.365	3.128	99.4	9.8	5.68	5.01	3.00
9	4,7	9.365	3.128	99.4	9.8	5.68	5.01	3.00
10	1,4,6,7,10	0.733	0.223	99.4	10.5	3.98	4.59	3.00

**Table 5:** Run in combination mode with the filtering variant, for the 5 most complete clusters in the serial group 2 case. Here, compared to Table 4, data have been cut at 3.5 Å resolution. All statistics have improved, while completeness and multiplicity both remains at reasonable values.

Cluster Number	Datasets Filtered	Rmeas	Rpim	Completeness (%)	Multi-Plicity	Resolution CC1/2	Resolution Mn(I/sd)	Resolution Max
4	6	0.366	0.158	99.0	5.4	4.39	4.98	3.50
8	7	2.875	0.927	99.5%	9.9	5.17	4.98	3.50
6	none	1.876	0.691	99.3	7.4	5.04	5.76	3.50
9	4,7	2.875	0.927	99.5	9.9	5.17	4.98	3.50
10	1,4,6,7,10	0.383	0.115	99.8	11.0	4.01	4.69	3.50

**Table 6:** Run in combination mode with the PRUNING variant, for the 5 datasets of Table 4. Statistics have improved in general. Values for the last row have not changed because the automated procedure has excluded no images.

Cluster Number	Datasets Filtered	Rmeas	Rpim	Completeness (%)	Multi-Plicity	Resolution CC1/2	Resolution Mn(I/sd)	Resolution Max
4	6	0.328	0.148	97.5	4.8	4.37	5.01	3.50
8	7	0.704	0.256	98.5	7.5	5.17	4.98	3.50
6	none	1.314	0.504	99.5	7.0	5.31	5.84	3.50
9	4,7	0.704	0.256	98.5	7.5	6.07	4.96	3.50
10	1,4,6,7,10	0.383	0.115	99.8	11.0	4.01	4.69	3.50

**Table 7:** Final overall refinement statistics for the two datasets used in this paper.

Dataset	Resolution Low (Å)	Resolution High (Å)	Completeness (%)	Rwork	Rfree
serial25_01.mtz	98.00	3.80	91.87	0.36	0.42
serial02_01.mtz	104.49	3.50	89.71	0.41	0.51

## References

[1] Liu Q, Zhang Z, Hendrickson WA (2011). Multi-crystal anomalous diffraction for low-resolution macromolecular phasing. Acta Cryst.

[2] Giordano R, Leal RMF, Bourenkov GP, McSweeney S, Popov AN (2012). The application of hierarchical cluster analysis to the selection of isomorphous crystals. Acta Cryst.

[3] Foadi J, Aller P, Alguel Y, Cameron A, Axford D, Owen RL, Armour W, Waterman DG, Iwata S, Evans G (2013). Clustering procedures for the optimal selection of data sets from multiple crystals in macromolecular crystallography. Acta Cryst.

[4] Barends TRM, Foucar L, Shoeman RL, Bari S, Epp SW, Hartmann R, Hauser G, Huth M, Kieser C, Lomb L (2013). Anomalous signal from S atoms in protein crystallographic data from an X-ray free-electron laser. Acta Cryst.

[5] White TA, Barty A, Stellato F, Holton JM, Kirian RA, Zatsepin NA, Chapman HN (2013). Crystallographic data processing for free-electron laser sources. Acta Cryst.

[6] El Omari K, Iourin O, Kadlec J, Fearn R, Hall DR, Harlos K, Grimes JM, Stuart DI (2014). Pushing the limits of sulfur SAD phasing: de novo structure solution of the N-terminal domain of the ectodomain of HCV E1. Acta Cryst.

[7] Liu Q, Guo Y, Chang Y, Cai Z, Assur Z, Mancia F, Greene MI, Hendrickson WA (2014). Multi-crystal native SAD analysis at 6 keV. Acta Cryst.

[8] Akey DL, Brown WC, Konwerski J, Ogata CM, Smith JL (2014). Use of massively multiple merged data for low-resolution S-SAD phasing and refinement of flavivirus NS1. Acta Cryst.

[9] Zander U, Bourenkov G, Popov A, de Sanctis D, Svensson O, McCarthy A, Round E, Gordeliy V, Mueller-Dieckmann C, Leonard GA (2015). MeshAndCollect: An automated multi-crystal data-collection workflow for synchrotron macromolecular crystallography beamlines. Acta Cryst.

[10] Rose J, Wang B-C, Weiss MS (2015). Native SAD is maturing. lUCrJ.

[11] Li D, Pye VE, Caffrey M (2015). Experimental phasing for structure determination using membrane-proteincrystals grown by the lipid cubic phase method. Acta Cryst.

[12] Axford D, Foadi J, Hu N-J, Choudhury H, Iwata S, Beis K, Evans G, Yilmaz A (2015). Structure determination of an integral membrane protein at room temperature from crystals in situ. Acta Cryst.

[13] Schlichting I (2015). Serial femtosecond crystallography: The first five years. IUCrJ.

[14] Olieric V, Weinert T, Finke D, Anders C, Li D, Olieric N, Borca C, Steinmetz M, Caffrey M, Jinek M (2016). Data-collection strategy for challenging native SAD phasing. Acta Cryst.

[15] Akey DL, Terwilliger TC, Smith JL (2016). Efficient merging of data from multiple samples for determination of anomalous substructures. Acta Cryst.

[16] Liu Q, Dahmane T, Zhang Z, Assur Z, Brasch J, Shapiro L, Mancia F, Hendrickson WA (2012). Structures from anomalous diffraction of native biological macromolecules. Science.

[17] Weinert T, Olieric V, Waltersperger S, Panepucci E, Chen L, Zhang H, Zhou D, Rose J, Ebihara A, Kuramitsu S (2015). Fast native S-SAD phasing for routine macromolecular structure determination. Nat Methods.

[18] Krojer T, Talon R, Pearche N, Collins P, Douangamath A, Brandao-Neto J, Dias A, Marsden B, von Delft F (2017). The XChemExplorer graphical workflow tool for routine or large-scale protein-ligand structure determination. Acta Cryst.

[19] Pearce N, Krojer T, Bradley A, Collins P, Nowak R, Talon R, Marsden B, Kelm S, Shi J, Deane CM (2017). A multi-crystal method for extracting obscured crystallographic states from conventionally uninterpretable electron density. Nat Commun.

[20] Roessler C, Kuczewski A, Stearns R, Ellson R, Olechno J, Orville A, Allaire M, Soares AS, Heroux A (2013). Acoustic methods for high-throughput protein crystal mounting at next-generation macromolecular crystallographic beamlines. J Synchrotron Rad.

[21] Axford D, Owen R, Aishima J, Foadi J, Morgan A, Robinson J, Nettleship J, Owens R, Moraes I, Fry E (2012). In situ macromolecular crystallography using microbeams. Acta Cryst.

[22] Lobley CMC, Sandy J, Sanchez-Weatherby J, Mazzorana M, Krojer T, Nowak RP, Sorensen TL (2016). A generic protocol for protein crystal dehydration using the HC1b humidity controller. Acta Cryst.

[23] Delageniere S, Brenchereau P, Launer L, Ashton A, Leal R, Veyrier S, Gabadinho J, Gordon E, Jones S, Levik KE (2011). ISPyB: An information management system for synchrotron macromolecular crystallography. Bioinformatics.

[24] Welcome to VMXi. http://www.diamond.ac.uk/Beamlines/Mx/VMXi.html.

[25] Chapman H, Barty A, Bogan M, Boutet S, Frank M, Hau-Riege P, Marchesini S, Woods B, Bajt S, Benner WH (2006). Femtosecond diffraction imaging with a soft-X-ray free-electron laser. Nat Phys.

[26] Barends TRM, Foucar L, Botha S, Doak R, Shoeman R, Nass K, Koglin J, Williams G, Boutet S, Messerschmidt M (2014). De novo protein crystal structure determination from X-ray free-electron laser data. Nature.

[27] Fromme P, Spence JCH (2011). Femtosecond nanocrystallography using X-ray lasers for membrane protein structure determination. Curr Opin Struct Biol.

[28] Gati C, Bourenkov G, Klinge M, Rehders D, Stellato F, Oberthür D, Yefanov O, Sommer B, Mogk S, Duszenko M (2014). Serial crystallography on in vivo grown microcrystals using synchrotron radiation. IUCrJ.

[29] Stellato F, Oberthür D, Liang M, Bean R, Gati C, Yefanov O, Barty A, Burkhardt A, Fischer P, Galli L (2014). Room-temperature macromolecular serial crystallography using synchrotron radiation. IUCrJ.

[30] Botha S, Nass K, Barends TRM, Kabsch W, Latz B, Dworkowski F, Foucar L, Panepucci E, Wang M, Shoeman R (2015). Room-temperature serial crystallography at synchrotron X-ray sources using slowly flowing free-standing high-viscosity microstreams. Acta Cryst.

[31] Coquelle N, Brewster A, Kapp U, Shilova A, Weinhausen B, Burghammer M, Colletier J (2015). Raster-scanning serial protein crystallography using micro- and nano-focused synchrotron beams. Acta Cryst.

[32] Diederichs K, Karplus PA (2013). Better models by discarding data?. Acta Cryst.

[33] Assmann G, Brehm W, Diederichs K (2016). Identification of rogue datasets in serial crystallography. J Appl Cryst.

[34] Hanson M, Roth C, Jo E, Griffith M, Scott F, Reinhart G, Desale H, Clemons B, Cahalan S, Schuerer S (2012). Crystal structure of a lipid G protein-coupled receptor. Science.

[35] Jain A, Murty M, Flynn PJ (1999). Data clustering: A review. ACM Comput Surv.

[36] Evans P (2006). Scaling and assessment of data quality. Acta Cryst.

[37] Evans G, Murshudov G (2013). How good are my data and what is the resolution?. Acta Cryst.

[38] Foadi J, Aller P BLEND: Managing, Scaling and Merging Multiple Datasets. http://www.ccp4.ac.uk/tutorials/tutorial_files/blend_tutorial/BLEND_tutorial.html.

[39] Aller P, Geng T, Evans G, Foadi J (2016). Applications of the BLEND Software to Crystallographic Data from Membrane Proteins. The Next Generation in Membrane Protein Structure Determination.

[40] Diamond Light Source. http://www.diamond.ac.uk.

[41] Olson E, Nordheim A (2010). Linking actin dynamics and gene transcription to drive cellular motile functions. Nat Rev Mol Cell Biol.

[42] Posern G, Treisman R (2006). Actin' together: serum response factor, its cofactors and the link to signal transduction. Trends Cell Biol.

[43] Pellegrini L, Tan S, Richmond T (2002). Structure of serum response factor core bound to DNA. Nature.

[44] Hassler M, Richmond T (2001). The B-box dominates SAP-1-SRFinteractions in the structure of the ternary complex. J EMBO.

[45] Zaromytidou A, Miralles F, Treisman R (2006). MAL ternary complex factor use different mechanisms to contact a common surface on the serum response factor DNA-binding domain. Mol Cell Biol.

[46] R Core Team R: A language and environment for statistical computing. https://www.R-project.org.

[47] Berman HM, Westbrook J, Feng Z, Gilliland G, Bhat TN, Weissig H, Shindyalov IN, Bourne PE (2000). The Protein Data Bank. Nucleic Acids Res.

[48] McCoy AJ, Grosse-Kunstleve RW, Adams PD, Winn MD, Storoni LC, Read RJ (2007). Phaser crystallographic software. J Appl Cryst.

[49] Emsley P, Cowtan K (2004). Coot: model-building tools for molecular graphics. Acta Cryst.

[50] Murshudov GN, Vagin AA, Dodson EJ (1997). Refinement of macromolecular structures by the maximum-likelihood method. Acta Cryst.

[51] Douangamath A, Aller P, Sanchez-Wheatherby J, Moraes I, Brandao-Neto J (2013). Using high-throughput in situ plate screening to evaluate the effect of dehydration on protein crystals. Acta Cryst.

[52] Cowtant K CSYMMATCH. http://www.ccp4.ac.uk/html/csymmatch.html.

[53] Dodson E CCP4 Bullettin Board. https://www.jiscmail.ac.uk/cgi-bin/webadmin?A2=ind1707&L=ccp4bb&0=A&P=7301.

