# Supplementary material for: A Novel Approach to Data Collection for Difficult Structures: Data Management for Large Numbers of Crystals with the BLEND Software
Source: Crystals (Basel). Author manuscript; Available in PMC 2018 Feb 15. (PMC5813789; doi:10.3390/cryst7080242)
Supplement: Appendix [file NIHMS76039-supplement-Appendix.pdf]

Appendix A

Crystals and X-ray data for the case described in this paper were produced and collected over a period of 2 years. Full details are included in Table A1. In the table, *Visit ID* refers to the unique code assigned by the Diamond synchrotron user office to the specific experiment at a given beamline.

The crystal used for data collection is named in the *Crystal* column. It is stored in liquid nitrogen inside a *Puck*, containing several crystals. The crystal itself can be large enough for the beam to be shone through it at several *Positions*. The *Serial Number*, thus, assigns a unique number to all sweeps collected for this structure. The remaining 5 columns describe how the crystal was prepared, including details of the presence of a soaked or co-crystallised heavy atom, and cooled down to cryo-temperatures. There are three *Base Conditions*: bc1, bc2 and bc3. They are a mixture of commercial screens, additive screens (not revealed, as they are sensitive data) and gadolinium (Gd). More specifically:

(1) bc1 = A + B + C1
(2) bc2 = A + B + C2
(3) bc3 = A + B + C1 + Gd

where,

A = commercial screen
B = commercial screen for optimization
C1 = additive screen
C2 = additive screen
Gd = gadolinium

There are also three types of *Cryogenic Conditions*:

(1) cry1 = 30% glycerol + 5 mM magnesium chloride
(2) cry2 = 30% glycerol + OH
(3) cry3 = 30% glycerol + 5 mM magnesium chloride + 1 M sodium bromide

Also, some crystals have been dehydrated by addition of salts directly in the crystallization plates [51] with one of two protocols, dh1 or dh2 (column *Dehydration*).

Table A1. Information on all datasets used for the work described in this paper. Crystals were prepared with one of three different base conditions, bc1, bc2 and bc3 (see text). They were also prepared for cooling with one of three different cryogenic conditions, cry1, cry2 and cry3 (see text). The majority of crystals included heavy atoms to attempt SAD phasing. Heavy atoms were soaked in solution for most of the crystals; in a few cases, they were co-crystallised. In order to improve resolution, one of two dehydration screenings have been attempted for many crystals. The table also lists details concerning dates of the various data collections, position of the crystals in the pucks and whether crystals were shot once or more times. The serial number, thus, corresponds to a unique and specific sweep obtained from X-ray diffraction.

Date	Visit ID	Puck	Crystal	Position	Serial N.	Base Condition	Cryogenic Condition	Dehydration	Co-Crystallized	Heavy Atom
02/05/2013	mx8031-26	777	xtal1	1	1	bc1	cry2	no	no	no
				2	2					
				3	3					
				4	4					
			xtal3	1	5	bc1	cry2	no	no	no
				2	6					
				3	7					
				4	8					
				5	9					
			xtal6	1	10	bc1	cry2	no	no	no
				2	11					
				3	12					
			xtal8	1	13	bc1	cry2	no	no	no
				b_1	14					
22/05/2013	mx8681-3	777	xtal3	real_3_2	15	bc1	cry1	no	no	no
				real_3c_2	16					
				real_3d_3	17					
				real_3e_2	18					
			xtal4	1	19	bc1	cry1	no	no	no
			xtal6	6_1	20	bc1	cry1	no	no	no
				6a_2	21					
				6b_1	22					
				6b_2	23					
				6c_1	24					
			xtal9	9_1	25	bc2	cry1	no	no	no
				9b_1	26					
				9b_2	27					
				9b_3	28					
			xtal14	b_1	29	bc2	cry1	no	no	no
		778	xtal3	3_2	30	?	?	?	?	?

Table A1. *Cont.*

Date	Visit ID	Puck	Crystal	Position	Serial N.	Base Condition	Cryogenic Condition	Dehydration	Co-Crystallized	Heavy Atom
20/10/2013	mx8681-13	542	1_13	3	31	bc1	cry1	dh1	no	no
				5	32					
				6	33					
				7	34					
				8	35					
				9	36					
				10	37					
13/02/2014	mx5005-1	136	7	7	38	bc1	cry1	dh1	no	$KICl_6$
				7b	39					
			8	8	40	bc1	cry1	dh1	no	$KICl_6$
				8b	41					
			10	10	42	bc1	cry1	dh1	no	$KICl_6$
				10b	43					
		138	xtal15	15_1	44	bc1	cry1	dh1	no	Tantalum
		542	xtal2	1	45	bc1	cry1	dh1	no	Hg (Thi)
			xtal4	1	46	bc1	cry1	dh1	no	Hg (Thi)
		544	xtal2	_	47	bc1	cry1	dh1	no	Pt (PIP)
		546	xtal4	4_1	48	bc1	cry1	dh1	yes	$KAu(CN)_2$
		758	xtal1	1	49	bc1	cry1	dh1	no	Hg (Ace)
			xtal9	1	50	bc1	cry1	dh1	no	Hg (Thi)
		762	xtal1	2	51	bc1	cry1	dh1	no	K_2PtCl_4
			xtal2	data	52	bc1	cry1	dh1	no	K_2PtCl_4
			xtal4	1	53	bc1	cry1	dh1	no	K_2PtCl_4
			xtal13	1	54	bc1	cry1	dh1	no	$KAu(CN)_2$
			xtal14	1	55	bc1	cry1	dh1	no	$KAu(CN)_2$
			xtal15	1	56	bc1	cry1	dh1	no	$KAu(CN)_2$
		764	xtal14	3	57	bc1	cry1	no	no	no
				4	58					
				5	59					
		765	xtal5	5_1	60	bc1	cry1	dh1	no	Hg (PMA)
		766	xtal3	3_1	61	bc1	cry1	dh1	no	K_2PtI_6

Table A1. *Cont.*

Date	Visit ID	Puck	Crystal	Position	Serial N.	Base Condition	Cryogenic Condition	Dehydration	Co-Crystallized	Heavy Atom
17/02/2014	cm4982-1	CPS-0134	12	2	62	bc1	cry1	dh1	yes	$OsCl_3$
			13	2<br>3<br>4	63<br>64<br>65	bc1	cry1	dh1	yes	K_2PtCl_4
		CPS-0140	7<br>11<br>12<br>15	7_1<br>11_4<br>12_1<br>15_1	66<br>67<br>68<br>69	bc1<br>bc1<br>bc1<br>bc1	cry1<br>cry1<br>cry1<br>cry1	dh1<br>dh1<br>dh1<br>dh1	yes<br>yes<br>yes<br>yes	K_2PtCl_4<br>Pt (PIP)<br>AgN<br>I3C (m.triangle)
		CPS-0761	1	2	70	bc1	cry1	dh1	yes	$GdCl_3$
			2	2<br>line	71<br>72	bc1	cry1	dh1	yes	$GdCl_3$
			5	1<br>2<br>5	73<br>74<br>75	bc1	cry1	dh1	yes	$GdCl_3$
			7	1	76	bc1	cry1	dh1	yes	$GdCl_3$
			9	2<br>3	77<br>78	bc1	cry1	dh1	yes	$GdCl_3$
02/05/2014	cm4982-2	767	data_0767_2	2<br>3<br>4<br>9<br>10<br>13<br>15	79<br>80<br>81<br>82<br>83<br>84<br>85	bc1	cry1	dh1	no	$KPtCl_4$
			data_0767_7	1<br>2<br>3<br>4<br>5<br>6<br>7<br>8<br>9<br>10<br>11<br>12<br>14<br>15<br>16	86<br>87<br>88<br>89<br>90<br>91<br>92<br>93<br>94<br>95<br>96<br>97<br>98<br>99<br>100	bc1	cry1	dh1	no	$KPtCl_4$

Table A1. *Cont.*

Date	Visit ID	Puck	Crystal	Position	Serial N.	Base Condition	Cryogenic Condition	Dehydration	Co-Crystallized	Heavy Atom
02/05/2014	cm4982-2	767	data_0767_9	1	101	bc1	cry1	dh1	no	$KPtCl_4$
				3	102					
				4	103					
				5	104					
				6	105					
			data_0767_10	1	106	bc1	cry1	dh1	no	$KPtCl_4$
				2	107					
				3	108					
				4	109					
				5	110					
				6	111					
				7	112					
				11	113					
			data_0767_11	1	114	bc1	cry1	dh1	no	$KPtCl_4$
				2	115					
				3	116					
				4	117					
				5	118					
				7	119					
				8	120					
				9	121					
				10	122					
				11	123					
			data_0767_13	2	124	bc1	cry1	dh1	no	$KPtCl_4$
				3	125					
				6	126					
			data_0767_14	1	127	bc1	cry1	dh1	no	$KPtCl_4$
				2	128					
				3	129					
				4	130					
			data_0767_15	1	131	bc1	cry1	dh1	no	$KPtCl_4$
				2	132					
				3	133					
				4	134					
		754	1	1_1	135	bc1	cry3	dh2	no	$KPtCl_4$
				1_2	136					
				1_3	137					
				1_4	138					
				1_5	139					
				1_6	140					
				1_7	141					
				1_8	142					
				1_9	143					
				1_10	144					

Table A1. *Cont.*

Date	Visit ID	Puck	Crystal	Position	Serial N.	Base Condition	Cryogenic Condition	Dehydration	Co-Crystallized	Heavy Atom
02/05/2014	cm4982-2	754	4	4_1	145	bc1	cry3	dh2	no	$KPtCl_4$
				4_2	146					
				4_3	147					
				4_4	148					
				4_5	149					
				4_6	150					
				4_7	151					
				4_8	152					
				4_9	153					
				4_10	154					
				4_11	155					
			5	5_1	156	bc1	cry3	dh2	no	$KPtCl_4$
				5_2	157					
				5_3	158					
		758	02	2_2	159	bc3	cry1	dh1	no	Os
				2_3	160					
			03	3_1	161	bc3	cry1	dh1	no	Os
			04	4_2	162	bc3	cry1	dh1	no	Os
				4_4	163					
				4_5	164					
			05	5_1	165	bc3	cry1	dh1	no	Os
				5_2	166					
				5_3	167					
				5_4	168					
				5_5	169					
			06	6_1	170	bc3	cry1	dh1	no	Os
				6_2	171					
				6_3	172					
				6_4	173					
				6_5	174					
			08	8_1	175	bc3	cry1	dh1	no	Os
			10	10_1	176	bc3	cry1	dh1	no	Os
				10_2	177					
			11	11_1	178	bc3	cry1	dh1	no	Os
				11_2	179					
			13	13_1	180	bc3	cry1	dh1	no	Os
				13_2	181					
			15	15_2	182	bc3	cry1	dh1	no	Os
				15_3	183					
				15_4	184					
				15_5	185					
				15_6	186					

Table A1. *Cont.*

Date	Visit ID	Puck	Crystal	Position	Serial N.	Base Condition	Cryogenic Condition	Dehydration	Co-Crystallized	Heavy Atom
02/05/2014	cm4982-2	765	1	1_1<br>1_3<br>1_4<br>1_5<br>1_6<br>1_7<br>1_8<br>1_9<br>1_10<br>1_11<br>1_12	187<br>188<br>189<br>190<br>191<br>192<br>193<br>194<br>195<br>196<br>197	bc3	cry1	dh1	no	$KPtCl_4$
			2	2_3<br>2_4<br>2_5<br>2_6<br>2_7<br>2_8<br>2_9<br>2_10<br>2_11<br>2_12<br>2_13<br>2_14	198<br>199<br>200<br>201<br>202<br>203<br>204<br>205<br>206<br>207<br>208<br>209	bc3	cry1	dh1	no	$KPtCl_4$
			5	5_4<br>5_6<br>5_7<br>5_8<br>5_9	210<br>211<br>212<br>213<br>214	bc1	cry1	dh1	no	Os
30/06/2014	cm4982-3	758	0758_3	3_1<br>3_2<br>3_3<br>3_4	215<br>216<br>217<br>218	bc1	cry1	dh2	no	$KPtCl_4$
			0758_4	4_1	219	bc3	cry1	dh2	no	Os

Table A1. *Cont.*

Date	Visit ID	Puck	Crystal	Position	Serial N.	Base Condition	Cryogenic Condition	Dehydration	Co-Crystallized	Heavy Atom
30/06/2014	cm4982-3	758	0758_5	5_1	220	bc3	cry1	dh2	no	Os
				5_2	221					
				5_3	222					
				5_4	223					
				5_5	224					
				5_6	225					
				5_7	226					
				5_8	227					
				5_9	228					
				5_10	229					
				5_11	230					
				5_12	231					
				5_13	232					
				5_14	233					
			0758_6	6_1	234	bc3	cry1	dh2	no	Os
				6_2	235					
				6_4	236					
				6_5	237					
				6_6	238					
			0758_8	8_1	239	bc3	cry1	dh2	no	Os
				8_2	240					
				8_3	241					
			0758_9	9_1	242	bc3	cry1	dh2	no	Os
				9_2	243					
				9_3	244					
				9_4	245					
				9_5	246					
				9_6	247					
				9_7	248					
			0758_10	10_1	249	bc3	cry1	dh2	no	Os
				10_2	250					
				10_3	251					
				10_4	252					
				10_5	253					
				10_6	254					
			0758_11	11_1	255	bc3	cry1	dh2	no	Os
				11_2	256					
				11_3	257					
				11_4	258					
				11_5	259					
				11_6	260					

Table A1. *Cont.*

Date	Visit ID	Puck	Crystal	Position	Serial N.	Base Condition	Cryogenic Condition	Dehydration	Co-Crystallized	Heavy Atom
30/06/2014	cm4982-3	758	0758_12	12_1<br>12_2<br>12_3<br>12_4<br>12_5<br>12_6<br>12_7	261<br>262<br>263<br>264<br>265<br>266<br>267	bc1	cry1	dh2	no	Os
			0758_13	line	268	bc1	cry1	dh2	no	Os
			0758_14	14_1<br>14_2	269<br>270	bc1	cry1	dh2	no	Os
			0758_15	15_1	271	bc1	cry1	dh2	no	Os

Appendix B

A table different from Table A1, but related to it, is Table 1, included in Section 4.3. Table 1 is a representation of a reshaped dataframe, an object present in the *R programming language* [46]. In this appendix, it will be explained how the reshaped dataframe is obtained. The starting point is the manual construction of a dataframe associated with Table A1. Several solutions can be envisaged to avoid this time-consuming task, all of them making use of database algorithms. These will be implemented shortly in *BLEND*, but for the work described in this article, preparation of the initial dataframe and the subsequent formation of the reshaped dataframe were carried out manually. A few lines of the code for the initial dataframe are shown in Figure A1.

	Date	VisitID	Puck	Crystal	Position	BaseCondition	CryogenicCondition	Dehydration	CoCrystallization	HeavyAtom
1	02/05/2013	mx8031-26	777	xtal1	1	bc1	cry2	no	no	no
20	22/05/2013	mx8681-3	777	xtal6	6_1	bc1	cry1	no	no	no
50	13/02/2014	mx5005-1	758	xtal9	1	bc1	cry1	dh1	no	Hg(Thi)
130	02/05/2014	cm4982-2	767	data_0767_14	4	bc1	cry1	dh1	no	K2PtCl4
200	02/05/2014	cm4982-2	765	2	2_5	bc3	cry1	dh1	no	K2PtCl4
201	02/05/2014	cm4982-2	765	2	2_6	bc3	cry1	dh1	no	K2PtCl4
202	02/05/2014	cm4982-2	765	2	2_7	bc3	cry1	dh1	no	K2PtCl4
203	02/05/2014	cm4982-2	765	2	2_8	bc3	cry1	dh1	no	K2PtCl4
204	02/05/2014	cm4982-2	765	2	2_9	bc3	cry1	dh1	no	K2PtCl4
205	02/05/2014	cm4982-2	765	2	2_10	bc3	cry1	dh1	no	K2PtCl4

Figure A1. Initial R dataframe, corresponding to Table A1. Just a few lines of the dataframe are shown in this figure.

The dataframe is a simple matrix in which each row corresponds to a single dataset. As multiple datasets can be associated with a same *Date*, *VisitID*, *Puck*, etc., then values for these columns are, often, repeated. Next, a dataframe including all possible combinations from the unique conditions in the initial dataframe, is created. Let us call this dataframe *theoretical conditions dataframe*. It turns out that the base conditions (BC) comprise 3 unique values (bc1, bc2, bc3), the cryogenic conditions (CC) also comprise 3 unique values (cry1, cry2, cry3), the dehydration protocol includes 3 unique values (no = no dehydration, dh1, dh2), the co-crystallisation flag (CO) includes two values (yes, no), and the heavy atom types (HA) are 15 (no = no heavy atom, KlCl6, Tantalum, Hg(Thi), Pt(PIP), KAu(CN)2, Hg(Ace), K2PtCl4, Hg(PMA), K2PtI6, OsCl3, AgN, IC3(m_triangle), GdCl3, Os). The possible combinations from all values listed above are $3 \times 3 \times 3 \times 2 \times 15 = 810$. This means that the theoretical conditions dataframe has 810 rows. Not all possible combinations will be present in the data collected for this work, because the total number of datasets is 271. For this reason, the initial dataframe entries are matched against the theoretical conditions dataframe; the result of this comparison is the new dataframe, simply called *conditions dataframe*, shown in Table 1.

Appendix C

With molecular replacement, models are oriented and placed at specific locations of the unit cell. Two solutions from molecular replacement runs do not necessarily overlap, even if they correspond to the same correct structure. The reason for this is that the asymmetric units selected by the molecular replacement program could be different. Furthermore, the absolute location of the oriented molecule depends on where the unit cell origin has been placed. The origin can be selected arbitrarily to be compatible with the specific symmetry. Thus, to verify whether two molecules overlap, all symmetry equivalents of the molecules and all allowed origin shifts must be tried. Within the CCP4 group of programs, this task is carried out by the program *CSYMMATCH* [52]. The input consists of the two files containing the atomic coordinates of the two models to be compared; one is considered the moving model, the other the reference model. The output consists of a PDB file corresponding to the moving model, transformed to the closest possible location to the reference model still compatible with symmetry and allowed unit cell origin. To compute the RMSD between all atoms of the reference structure and all atoms of the moved structure, we have used the CCP4 program *COMPAR*. This is an old program with no related documentation on the official CCP4 website. Details on how to run

this program have been learned via the CCP4 Bulletin Board [53]. The value for the two structures discussed in this paper is RMSD = 0.773 Å.
